# Molecular level insight into non-bilayer structure formation in thylakoid membranes: a molecular dynamics study

**DOI:** 10.1007/s11120-025-01156-3

**Published:** 2025-06-11

**Authors:** Bence Fehér, Gergely Nagy, Győző Garab

**Affiliations:** 1 Nanobiophysics Research Group, HUN-REN Office for Supported Research Groups, Budapest, 1094 Hungary; 2 Institute of Biophysics and Radiation Biology, Semmelweis University, Budapest, 1094 Hungary; 3 Neutron Scattering Division, Oak Ridge National Laboratory, Oak Ridge, Tennessee 37831 USA; 4 Institute of Plant Biology, HUN-REN Biological Research Centre, Szeged, 6726 Hungary; 5 Department of Physics, Faculty of Science, University of Ostrava, Ostrava, 71000 Czech Republic

**Keywords:** Thylakoid membranes, Non-bilayer lipid, MGDG, Dehydration, Inverted hexagonal phase

## Abstract

In oxygenic photosynthetic organisms, the light reactions are performed by protein complexes embedded in the lipid bilayer of thylakoid membranes (TMs). The organization of the bulk lipid molecules into bilayer structures provide optimal conditions for the build-up of the proton motive force (pmf) and its utilization for ATP synthesis. However, the lipid composition of TMs is dominated by the non-bilayer lipid species monogalactosyl diacylglycerol (MGDG), and functional plant TMs, besides the bilayer, contain large amounts of non-bilayer lipid phases. Bulk lipids have been shown to be associated with lumenal, stromal-side and marginal-region proteins and proposed to play roles in the self-assembly and photoprotection of the photosynthetic machinery. Furthermore, it has recently been pointed out that the generation and utilization of pmf for ATP synthesis according to the ‘protet’ or protonic charge transfer model Kell (Biochim Biophys Acta Bioenerg 1865(4):149504, 2024), requires high MGDG content Garab (Physiol Plant 177(2):e70230, 2025). In this study, to gain better insight into the structural and functional roles of MGDG, we employed all atom and coarse-grained molecular dynamics simulations to explore how temperature, hydration levels and varying MGDG concentrations affect the structural and dynamic properties of bilayer membranes constituted of plant thylakoid lipids. Our findings reveal that MGDG promotes increased membrane fluidity and dynamic fluctuations in membrane thickness. MGDG-rich stacked bilayers spontaneously formed inverted hexagonal phases; these transitions were enhanced at low hydration levels and at elevated but physiologically relevant temperatures. It can thus be inferred that MGDG plays important roles in heat and drought stress mechanisms.

## Introduction

Oxygenic photosynthetic organisms convert light energy into chemical energy using their highly organized interwoven flat membrane vesicles, the thylakoid membranes (TMs). Plant TMs accommodate virtually all constituents of the molecular machinery responsible for the light reactions of photosynthesis. The generation of the proton-motive force (pmf) between the inner and outer aqueous phases of TMs, and its chemiosmotic utilization (Mitchell 1966) are largely based on the insulating capability of these membranes. This can be warranted by organizing their bulk lipid molecules into bilayer structures; bilayers constitute the basis of the ‘standard’ fluid-mosaic model of biological membranes (Singer and Nicolson 1972). In TMs, the requirement to organize the lipid molecules into bilayers is readily satisfied for their cylindrically shaped, bilayer-forming lipid species. However, these lipids constitute only about half of the lipid content of TMs: digalactosyl-diacylglycerol (DGDG, 30%), phosphatidylglycerol (PG, 5–12%) and sulfoquinovose-diacylglycerol (SQDG, 5–12%) (Boudière et al. 2014). In contrast, the major lipid species of TMs, monogalactosyl-diacylglycerol (MGDG, 50%), belongs to the family of non-bilayer lipids species (Williams 1998). These conically shaped lipid molecules, which feature larger area in the hydrocarbon tail region than in the polar head region, preferentially form inverted hexagonal HII phase or cubic phase lipid architectures (Williams 1998; Israelachvili 2011; Demé et al. 2014;Jouhet 2013).

It has also been thoroughly documented that functional plant TMs contain in large quantities non-bilayer lipid assemblies (Krumova et al. 2008; Garab et al. 2017, 2022). The main protein complexes - the two photosystems along with their antenna complexes, the cytochrome b6f complex and the ATP synthase – have been shown to be located in regions dominated by the bilayer phases, small-size ‘lipid inclusions’ between the supercomplexes. In close connection with these regions, non-bilayer lipid phases have been shown to be associated with proteins and/or polypeptides in the inner (lumenal) and outer (stromal) aqueous phases, as well as in the marginal regions of TMs (Garab et al. 2025). In general, the polymorphic lipid phase behavior of TMs is explained within the framework of the Dynamic Exchange 2 Model (DEM) (Garab et al. 2000, 2017, 2022). This model assumes dynamic equilibrium between the bilayer and non-bilayer lipid phases. It posits that the bilayer organization of the membranes are stabilized by the supercomplexes; and that the ‘excess’ amounts of lipids are segregated, due to the high non-bilayer propensity of TM lipids. This is a self-regulatory mechanism that safeguards the high protein-to-lipid ratios in these membranes. By this means, DEM extends the fluid mosaic membrane model of Singer and Nicolson (1972) (Singer and Nicolson 1972) – by allowing the presence of non-bilayer lipid phases in functional TMs and adds an extra dimension to their structural dynamics and plasticity. It has also been shown that MGDG, as non-bilayer lipid, plays key role in important physiological functions, such as e.g. the operation of the photoprotective lumenal enzyme of violaxanthin deepoxidase (Latowski et al. 2002; Dlouhý et al. 2020); further, isotropic lipid phase have been inferred to be involved in membrane dynamics via mediating the fusion of membranes (Böde et al. 2024). Recently, it has been pointed out that the high abundance of MGDG, and the compromised membrane impermebaility of bilayers composed of TM lipids (Fehér et al. 2023), is not in conflict with the build up of pmf and its utilization for ATP synthesis (Garab et al. 2025). In fact, via warranting the high protein density of TMs, stabilizing the protein networks, and thus the contiguity of protonable protein residues on the lumenal side of TMs, require high MGDG concentrations. The high densitiy protein networks provide the structural basis required for the modified, surface-to-surface chemiosmotic mechanism (Goñi 2014; Kell 2024), as opposed to the ‘conventional’ bulk-water phase to bulk water phase mechanism (Mitchell 1966).

The high abundance of non-bilayer lipid molecules and high protein density of membranes are well known attributes of all energy converting biological membranes. Furthermore, in recent years it became clear that, similar to plant TMs, inner mitochondrial membranes (IMMs) of animal origin, capable of synthesizing ATP, contain substantial amounts of non-lamellar lipid phases (Gasanov et al. 2018; Garab et al. 2022). These findings highlight the complexity and highly dynamic nature of lipid organization within the two fundamental energy transducing membranes of the biosphere, TMs and IMMs. Nevertheless, our understanding concerning the roles of non-bilayer lipid molecules and non-lamellar lipid phases in the self-assembly and structural dynamics of TMs, and their correlations with the energization of the membranes and the utilization of pmf for ATP synthesis are still rudimentary and require further research. Molecular dynamics (MD) simulations for TM lipids have been performed during the past decade (van Eerden et al 2015) in focus or with the incorporation of selected protein components (Ogata et al. 2013; Van Eerden et al. 2017b, 2017, 2017a; Thallmair et al. 2019) and chlorophyll contents (Sahu et al. 2024). Earlier, we also studied the temperature dependent physical properties of these membranes with MD (Fehér et al. 2023).

Garg and Debnath (Garg and Debnath 2025) were the first to incorporate LHCII into stacked membrane simulations. Their results demonstrated that, in accordance with the experiments of Simidjiev et al. (Simidjiev et al. 2000), this protein inhibits non-bilayer formation, thereby acting as a regulator of membrane structure. LHCII, at low and moderate light intensity supplies excitation energy with high efficiency to the reaction center; in contrast, in high light, leading to a sustained acidification of the lumen, it dissipates excess excitation energy via non-photochemical quenching (NPQ) of the first excited singlet state of chlorophyll-a (Ruban et al. 2012). Recent MD simulations indicated the role of dynamic rearrangement of lipids around LHCII in this key regulatory function of plants (Wilson et al. 2024). Low pH induced reversible NPQ, associated with reorganizations in the lipid phases, detected by 31P-NMR spectroscopy, have earlier been observed in isolated spinach thylakoid membranes (Dlouhý et al. 2020).

Despite these advances, the physical and molecular mechanisms underlying the formation of non-bilayer structures and their structural and functional roles remain incompletely understood. In this study, we employ all-atom and coarse-grained molecular dynamics simulations to investigate the role of MGDG in bilayer membranes. Our findings reveal that MGDG exerts a local dehydrating effect on the bilayer and induces structural frustration, leading to significant thickness inhomogeneities and a decrease in lipid molecular order. Further, we demonstrate that, at low hydration state, even small amounts of MGDG in stacked membrane pairs can promote the formation of inverted hexagonal structures. The stability and dynamic properties of these structures are shown to be highly dependent on hydration levels and temperature and thus are likely to play crucial roles in drought and heat stress responses of plants. Our simulations also reveal lipid clustering, nanoscale inhomogeneities (van Eerden et al, 2015), within these molecular assemblies, underlining the importance of complex and dynamic nature of the bulk lipid molecules in regulatory processes of the photosynthetic machineries of plants.

## Methods

### Molecular dynamics simulations

All atom (AA) and coarse-grained (CG) molecular dynamics simulations were performed using GROMACS 2023.2 (Pronk et al. 2013). For all atom simulations the initial coordinates were built with CHARMM-GUI web server (Jo et al. 2008). The bilayers consisted lipids of seven type: 16:1(3 t)-16:0 and 16:1(3 t)18:3(9,12,15) PG, 18:3(9,12,15)-16:0 and di18:3(9,12,15) DGDG, 18:3(9,12,15)-16:0 and di18:3(9,12,15) MGDG and 18:3(9,12,15)-16:0 SQDG. The number ratios of the lipids were varied in such a way that the MGDG contents were 0, 5, 10, 20, 30, 40% and the ratio of the other lipids were corresponding to the lipid composition of thylakoid membranes. The composition of all atom simulations are listed in Table 1. The dimensions of the bilayer was approximately 10 nm in x and y direction. All simulations were performed in CHARMM36 force field (Klauda et al. 2010; Best et al. 2012). Sodium (Na$$^ + $$) and chloride (Cl$$^ - $$) ions were added to charge neutralize the system. Energy minimization was conducted for 5,000 steps using the steepest descent method. Equilibration were performed for 1,875 ps in three stages with integration time steps ranging from 1 fs to 2 fs. Long-range electrostatic interactions were calculated using the reaction field method with a cutoff distance of 1.2 nm (Tironi et al. 1995). Van der Waals interactions were treated with a cutoff of 1.2 nm. Temperature was controlled with the v-rescale thermostat (Bussi et al. 2007) with a 1 ps coupling time at 280, 300, and 320 K. Pressure was semi-isotropically coupled and maintained using the C-rescale barostat (Bernetti and Bussi 2020) with a 5 ps coupling time. Production runs were conducted for 300 ns with the same barostat parameters.

**Table 1:** System composition of all atom simulations

Molecule	0% MGDG	5% MGDG	10% MGDG	20% MGDG	30% MGDG	40% MGDG (Thylakoid)
16:1(3 t)18:3(9,12,15) PG	56	64	60	52	76	36
16:1(3 t)-16:0 PG	28	32	28	24	36	18
di18:3(9,12,15) MGDG	0	16	32	68	93	90
18:3(9,12,15)-16:0 MGDG	0	4	4	8	14	18
di18:3(9,12,15) DGDG	140	160	148	132	116	126
18:3(9,12,15)-16:0 DGDG	28	32	28	28	24	18
18:3(9,12,15)-16:0 SQDG	84	96	88	80	64	54
Water	15,551	17,277	17,818	16,738	16,748	17,403
Sodium ion	168	192	176	160	128	108

Coarse grained initial configurations of the bilayers were constructed using the insane.py script (Wassenaar et al. 2015). The dimensions of the simulation box were 20 nm in the x and y directions, while the z dimension was adjusted to achieve the desired hydration levels. Each system comprised approximately 1,400 lipids, including 16:1(3 t)-16:0 and 16:1(3 t)18:3 PG, 18:3(9,12,15)-16:0 and di18:3(9,12,15) DGDG, 18:3(9,12,15)-16:0 and di18:3(9,12,15) MGDG, and 18:3/16:0 SQDG lipids. The specific lipid compositions and notations are detailed in Table 2. The number of lipids was varied to achieve the desired MGDG content (0, 5, 10, 20, 30, 40%), while the ratios of the other lipids were kept constant. The water content in the simulation boxes was set to 8 and 12 water beads per lipid, corresponding approximately to 32 and 48 all-atom water molecules, respectively. The composition of each system is provided in Tables 3 and 4.

**Table 2:** Coarse-grained lipids in simulation box with notation used throughout the paper

Lipid type	Acyl chain	Notation
PG	16:1(3 t)/18:3	JFPG
	16:1(3 t)/16:0	JPPG
MGDG	di-18:3	DFMG
	18:3/16:0	FPMG
DGDG	di-18:3	DFGG
	18:3/16:0	FPGG
SQDG	18:3/16:0	FPSG

**Table 3:** System composition of coarse-grained simulations at hydration level of 8 water bead/lipid

Molecule	0% MGDG	5% MGDG	10% MGDG	20% MGDG	30% MGDG	40% MGDG (Thylakoid)
JFPG	226	214	202	182	156	134
JPPG	112	106	100	90	78	66
DFMG	0	58	118	220	354	472
FPMG	0	8	16	34	50	66
DFGG	562	534	506	456	394	338
FPGG	112	106	100	90	78	66
FPSG	338	320	304	274	236	202
Water	9959	10,087	10,170	10,327	10,312	10,276
Sodium ion	676	640	606	546	470	402

**Table 4:** System composition of coarse-grained simulations at hydration level of 12 water bead/lipid

Molecule	0% MGDG	5% MGDG	10% MGDG	20% MGDG	30% MGDG	40% MGDG (Thylakoid)
JFPG	226	214	202	180	156	134
JPPG	112	106	100	90	78	66
DFMG	0	58	118	236	354	472
FPMG	0	8	16	32	50	66
DFGG	562	534	506	450	394	338
FPGG	112	106	100	90	78	66
FPSG	338	320	304	270	236	202
Water	16,117	14,791	15,456	15,785	15,737	15,650
Sodium ion	676	640	606	540	470	402

Coarse-grained simulations utilized the Martini 2.2 force field (de Jong et al, 2013). Sodium (Na$$^ + $$) and chloride (Cl$$^ - $$) ions were added to neutralize the charge of the system. Structures were minimized for 5,000 steps using the steepest descent method, followed by 3,600 ps of equilibration in three stages with integration time steps ranging from 2 fs to 12 fs. Long-range electrostatic interactions were calculated using the reaction field method with a cutoff distance of 1.1 nm (Tironi et al. 1995). Van der Waals interactions were also treated with a cutoff of 1.1 nm. Temperature was controlled using the v-rescale thermostat (Bussi et al. 2007) with a 1 ps coupling time at 280, 300, and 320 K. Pressure was semi-isotropically coupled and maintained using the Parrinello-Rahman barostat (Parrinello and Rahman 1981) with a 12 ps coupling time. Production runs were conducted for 8 µs with the same barostat parameters. For the simulations of stacked bilayers, the final snapshot of the production run for the mono-bilayer systems was replicated in the z direction, and the simulation protocol was identical to that described above. Three independent simulations were performed for all systems. Visualization was carried out using PyMOL (The PyMOL Molecular Graphics System, version 2.0, Schrödinger, LLC).

### Analysis

#### Membrane thickness

Membrane thickness calculations for both CG and AA simulations were performed with Membrane Plugin, version 1.1 (Guixà-González et al. 2014) implemented in Visual Molecular Dynamics (VMD) (Humphrey et al. 1996). We identified the head groups as the phosphorous, the sulphuric and the ring oxygen atom in all atom simulations and phosphate bead (PO4) for JPPG and JFPG, the C1 carbon (part of the galactosyl group) for DFMG, FPMG, and FPSG, and the GA2 carbon (a more distal member of the galactosyl group) for FPGG and DFGG.

#### Second-rank order parameter

The second-rank order parameter (${P_2}$) (Tieleman et al. 1997; Marrink et al. 2004; Song et al. 2005; Chen et al. 2014; Piggot et al. 2017) was calculated individually for all lipid chains and subsequently averaged over all lipids and time. Thus, the ${P_2}$ values represent the overall ordering of lipid tails along the bilayer normal. The ${P_2}$ is defined as

1 $${P_2} = \frac{{\langle 3\mathop {\cos }\nolimits^2 \theta - 1\rangle }}{2}$$

where $\theta$ is the angle between the consecutive bonds and the bilayer normal. The angle brackets indicate molecular and temporal ensemble averages (Piggot et al. 2017). The ${P_2}$ was calculated for all atoms in the chain, which were indexed from 1 to 4, 1 meaning the atom closest to the headgroup while 4 means the atom most distant from the headgroup. According to this definition, ${P_2} = 1$ corresponds to perfect alignment with the bilayer normal, while ${P_2} = 0$ indicates random orientation, and ${P_2} = - 0.5$ represents anti-alignment. The ${P_2}$ was calculated and averaged for the last 4000 ns of the simulation.

#### Curvature

Mean average curvature was computed using a slightly modified version of the MDAnalysis MembraneCurvature Python toolkit (Michaud-Agrawal et al. 2011) to extract the maximum curvature of each frame.

#### Radial distribution functions

Radial distribution functions (rdf) were calculated with the Eq. 2

2 $${g_{AB}}(r) = \frac{1}{{{{\langle {\rho _B}\rangle }_{{\text{local}}}}}}\frac{1}{{{N_A}}}\sum\limits_i^{{N_A}} \sum\limits_j^{{N_B}} \frac{{\delta ({r_{ij}} - r)}}{{4\pi {r^2}}}$$

where $\langle {\rho _B}\rangle$ is density of particle $B$ within $r$ distance of particle $A$. ${\langle {\rho _B}\rangle _{{\text{local}}}}$ is the particle density of particle $B$ averaged over all spheres around particle $A$ within ${r_{max}}$. ${r_{max}}$ was defined as half of the box length.

#### Diffusion coefficients

The diffusion coefficient was determined by fitting a linear model to the mean-squared displacement (MSD) of the lipids as a function of time. In case of bilayers we calculated the MSD in 2 dimension, as described by Eq. 3.

3 $$MSD = 4Dt$$

where $MSD$ denotes the mean-squared displacement, $D$ is the diffusion coefficient, and $t$ represents time. Similarly, in case of inverted hexagonal structures we determined MSD for 3 dimensions, defined as

4 $$MSD = 6Dt$$

#### Contact matrices

Contacts were identified by considering all beads of each molecule. We calculated and averaged the number of contacts with beads of other molecules over the final 4000 nanoseconds of the CG simulation based on bead proximity. Two beads were considered to be in contact if their distance from each other was less than 0.5 nm. The number of contacts at the end of the simulation was normalized to the number of contacts at the initial configuration of the bilayer. Since the initial configuration can be approximated as a random distribution of lipids, a normalized contact number greater than 1 indicates a tendency for clustering, while a value less than 1 suggests a tendency for separation.

#### Statistical analysis

Statistical analysis was performed using pairwise t-tests in the R programming environment. These tests aimed to determine whether there were significant differences in thickness or curvature between each pair of compositions and temperatures. The pairwise.t.test() function from the R stats package was used for multiple pairwise comparisons of means with the Student’s t-test. The p-values were adjusted for multiple comparisons using the Bonferroni correction method to control for Type I error rate. This adjustment involves modifying the significance threshold to account for the number of comparisons performed, thereby reducing the likelihood of false positives. For each comparison, 95 % confidence intervals were computed to provide an estimate of the range within which the true difference in means is likely to fall, enhancing the interpretation of the statistical significance of the observed differences.

## Results and Discussion

### Bilayer properties

**Fig. 1:**
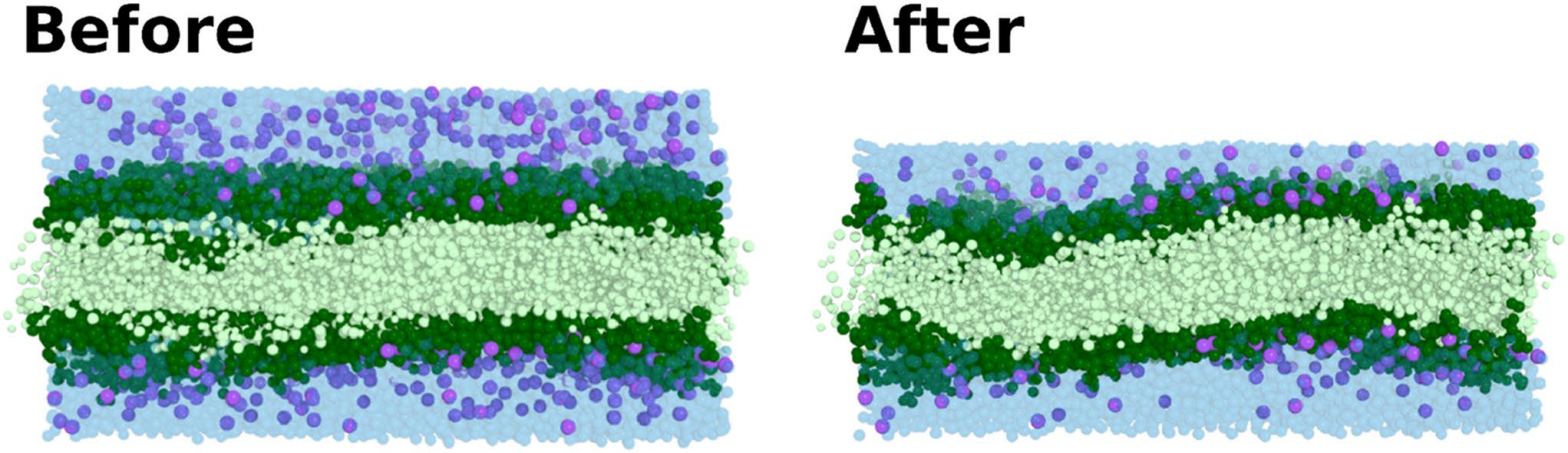
Snapshot of bilayer simulation with 40% MGDG at 280 K before and after the production run. Dark green, light green, light blue and purple indicates the headgroups, the tails of the lipids, the water and the ions respectively

We systematically characterized the properties of lipid bilayers as a function of increasing monogalactosyldiacylglycerol (MGDG) content, specifically at concentrations of 0, 5, 10, 20, 30, and 40%. Representative snapshots of initial and final configuration are displayed in Fig. 1. Initially, we focused on analysing the membrane thickness off all atom and coarse-grained simulations, defined by the spatial positioning of the head groups of the two leaflets.

**Fig. 2:**
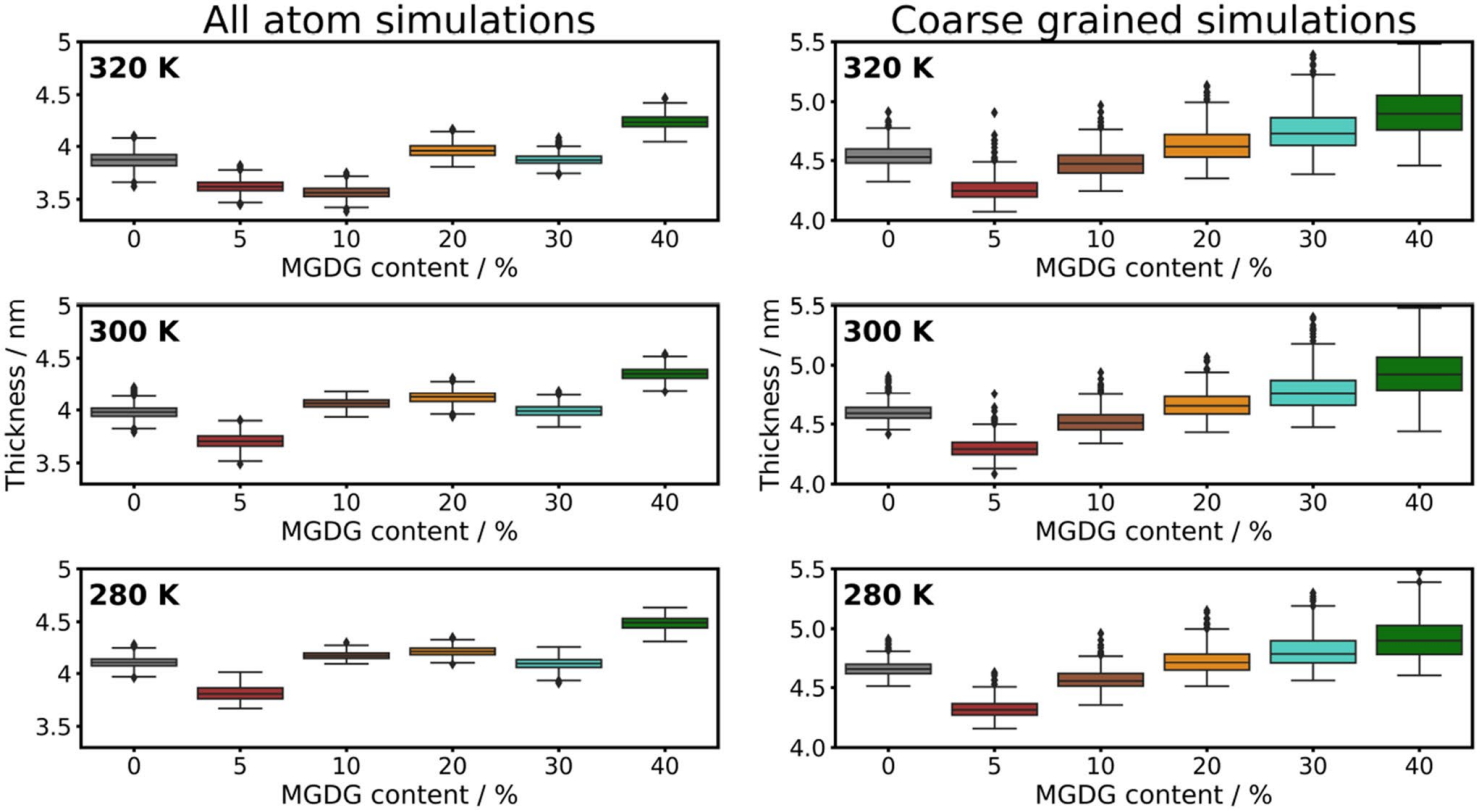
Thickness of bilayer at varying MGDG content at three different temperatures (three panels) obtained from all atom and coarse grained simulations

**Fig. 3:**
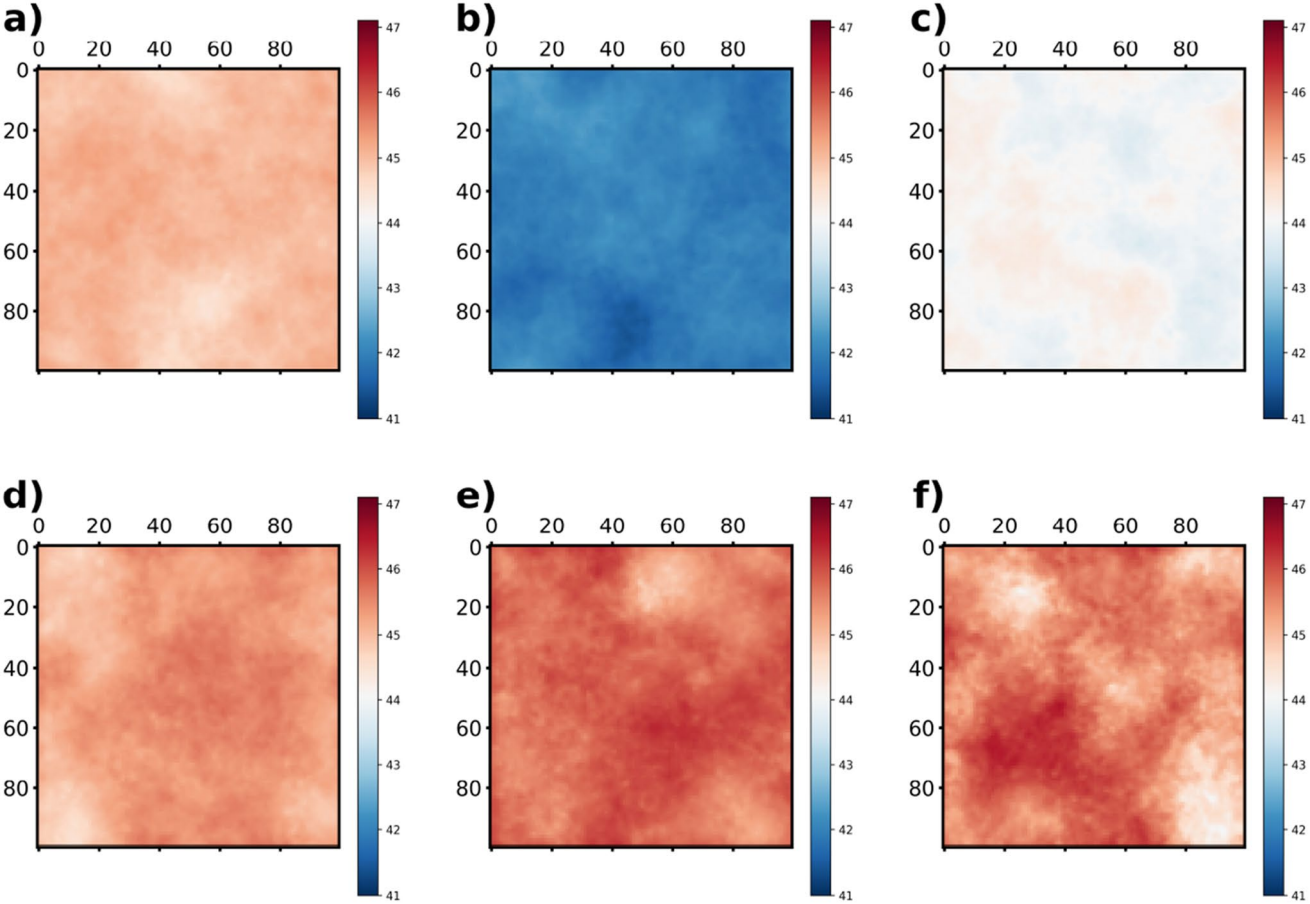
Thickness map of bilayer obtained from CG simulations at (**a**): 0 % (**b**): 5 % (**c**): 10 % (**d**): 20 % (**e**): 30 % and (**f**): 40 % MGDG content. All data are calculated from the simulations performed at 300 K

The thickness measurements of bilayers at each composition, recorded at three different temperatures, are illustrated in Fig. 2 both for all atom and coarse-grained simulations. Unlike conventional methods that display the average density profile along the bilayer normal over time, we calculated the thickness for each frame half of the simulation time (150 ns for AA and 4000 ns for CG), presenting the results using box plots. This methodology provides enhanced insight into the fluctuations of membrane thickness. Similar trends can be observed both for all atom and coarse-grained simulations. Notably, the incorporation of 5% MGDG into the bilayer significantly reduces the thickness compared to the MGDG-free bilayer. However, with the incremental addition of MGDG, there is a progressive increase in membrane thickness. This uncommon behaviour can most likely be explained by two phenomena. First, adding marginal amount of MGDG makes the bilayer more disorganized which decreases the thickness, however, increasing the MGDG content makes the bilayer more frustrated. Furthermore, a slight decrease in membrane thickness can be observed increasing the temperature in line with previous findings (Kučerka et al. 2011). To further elucidate the effect of MGDG on bilayer thickness, we generated thickness maps of CG simulations, as presented in Fig. 3. We created a grid of 100$\times$100 cells and collected the average z-values from the last 4000 ns of the simulation. The colorbars cover a range of 2.5 nm relative to the minimum values. Consequently, a more uniform color in the images indicates a more homogeneous membrane thickness. The results clearly show that the addition of a small amount of MGDG (5, 10 %) does not induce significant fluctuations in membrane thickness. However, increasing the MGDG content (20, 30, and 40 %) triggers the formation of thickness inhomogeneities, which become extreme at 40 % MGDG. This observation indicates that higher MGDG concentrations lead to increased inhomogeneity in membrane thickness.

**Fig. 4:**
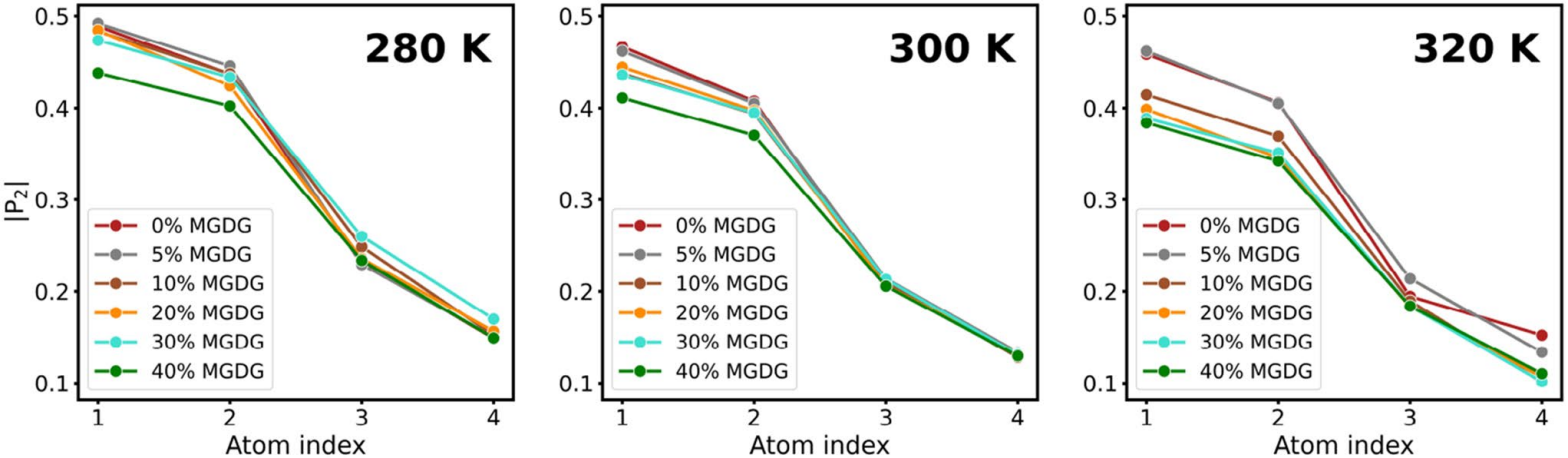
Second rank order parameter ($|{P_2}|$) of lipid molecules at varying MGDG content obtained from CG simulations at three different temperatures (three panel)

To elaborate on the effect of MGDG on the phase behavior of the thylakoid membrane at different temperatures, we calculated the absolute value of second-rank order parameter ($|{P_2}|$) (Tieleman et al. 1997; Song et al. 2005; Chen et al. 2014; Piggot et al. 2017). As shown in Fig. 4, at 280 K, there is no significant difference between the $|{P_2}|$ values for most samples, except for the 40% MGDG content, which indicates a lesser degree of ordering. At 300 K, the $|{P_2}|$ values start from a lower value compared to 280 K, and the effect of added MGDG is more pronounced, showing a gradual decrease in $|{P_2}|$ with increasing MGDG content. At 320 K, the $|{P_2}|$ values for 0 and 5% MGDG content do not differ significantly from those at 300 K. However, for MGDG content of 10% and above, there is a significant reduction in $|{P_2}|$. The relative ineffectiveness of MGDG at 280 K can be attributed to the fact that at this temperature, the membrane is near the freezing point of water, thereby reducing membrane dynamics. Conversely, at higher temperatures, the addition of MGDG decreases membrane order, resulting in increased fluidity.

**Fig. 5:**
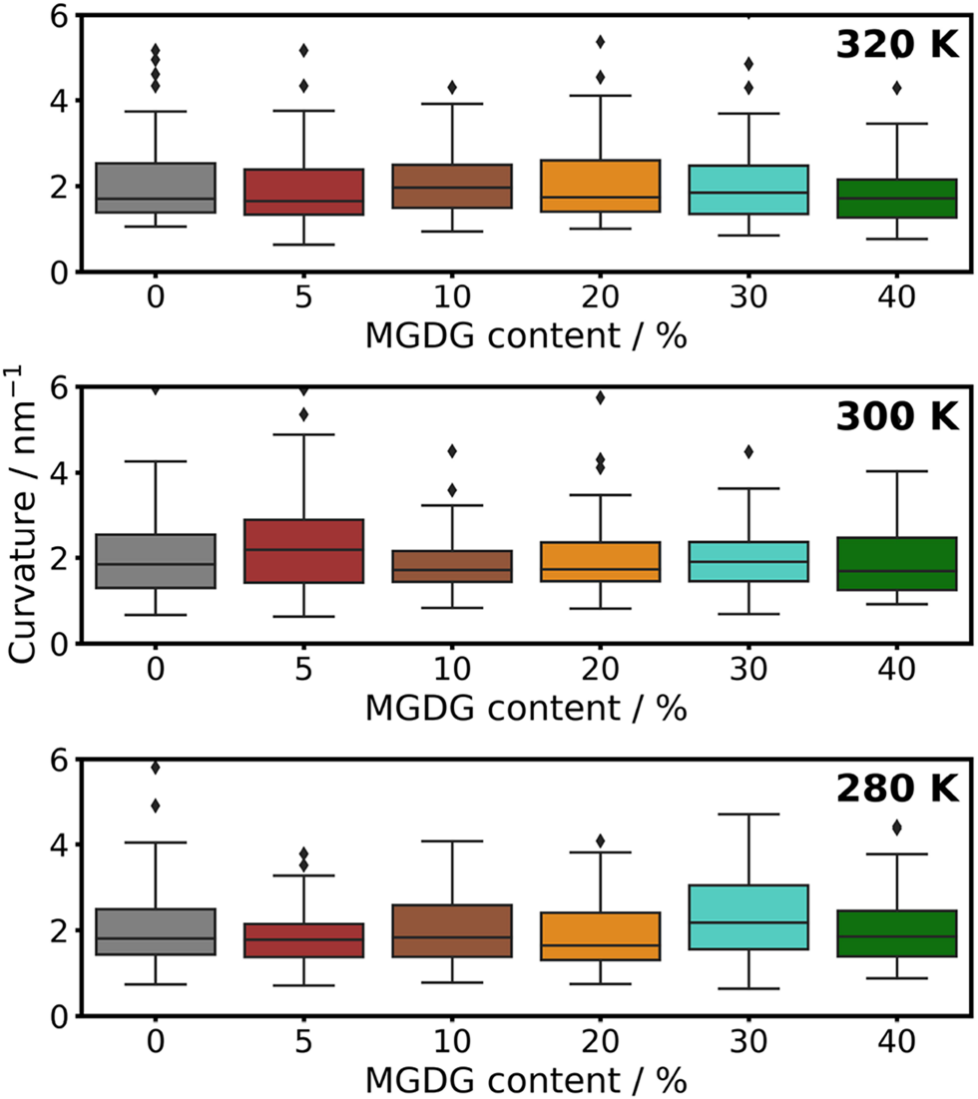
Mean curvature of lipid bilayer of different MGDG content as observed at different temperatures obtained from CG simulations

To determine if increasing amounts of MGDG induce changes in the average curvature of the thylakoid membrane, we calculated the mean curvature of the bilayers at each composition and temperature. For this calculation, we used a modified version of the MDAnalysis tool. Given the dynamic nature of the membrane and the absence of stabilizing components (e.g. proteins) or external forces, averaging the mean curvature over the entire trajectory would not be informative. Therefore, we determined the mean curvature for each configuration and represented the maximum values in a box plot. This method allows us to visually assess any trends in curvature. The results are presented in Fig. 5. It is clear that the bilayer even without MGDG has a slight (non-zero) curvature. However, according to the obtained p-values by pairwise t-test, there are no significant differences in curvature either between different compositions or between different temperatures.

**Fig. 6:**
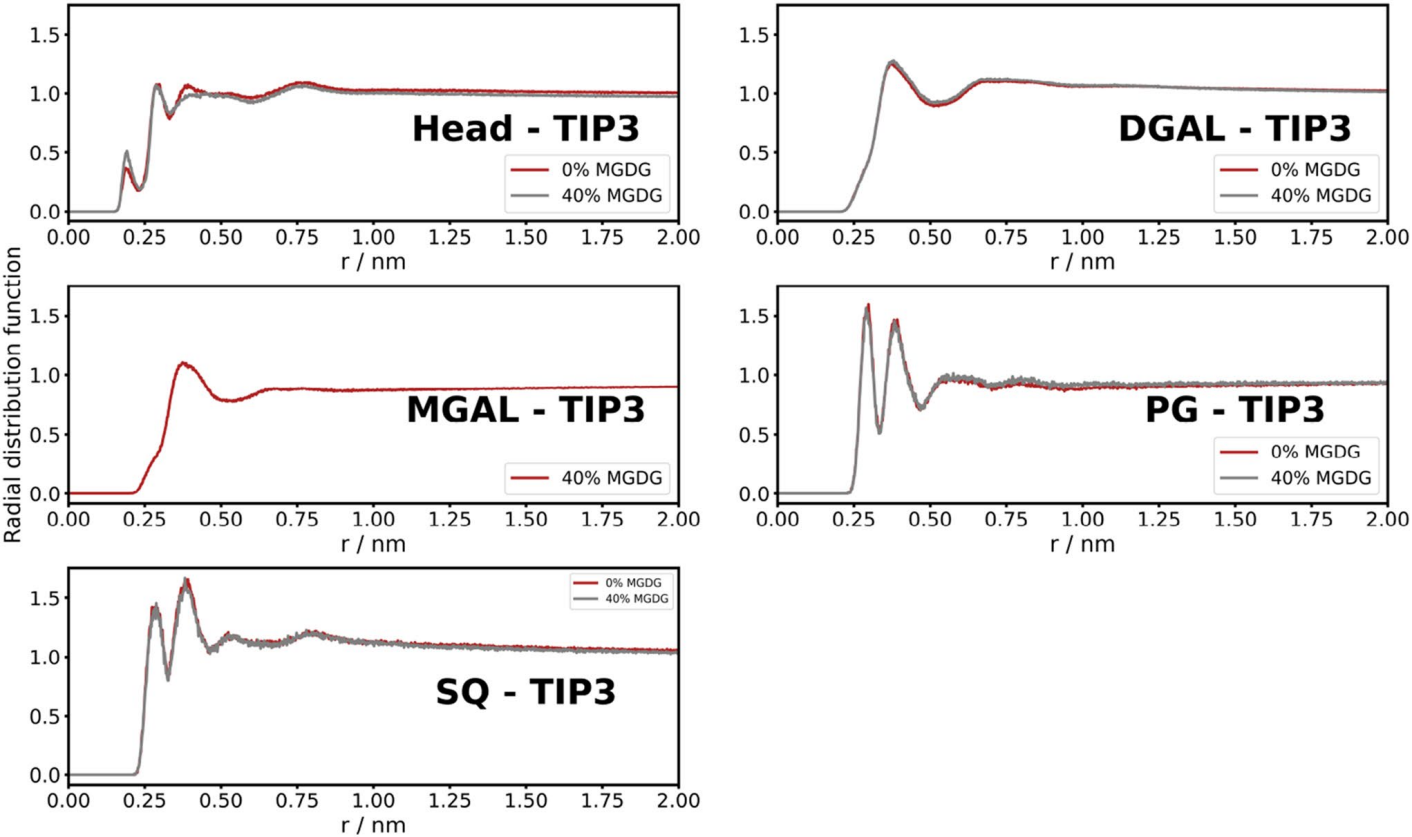
Radial distribution function of water (TIP3) around lipid headgroups defined as phosphatidylglycerol (PG), monogalactosyl (MGAL) of MGDG, digalactoysl (DGAL) of DGDG and sulfoquinovose (SQ) obtained from AA simulations

To investigate the water structure in the proximity of the bilayer, we calculated the radial distribution function (rdf) from all atom simulations. We calculated the distribution of water molecules (TIP3) around the headgroups and we also broke it down to phosphatidylglycerol (PG), monogalactosyl (MGAL), digalactosyl (DGAL) and sulfoquinovose (SQ) groups. The rdfs for 0 and 40 % MGDG are presented in Fig. 6. MGDG contains monogalactosyl and DGDG dicontains galactosyl groups, meaning that the addition of MGDG increases the galactosyl content of the membrane. It can be seen that for PG and SQ, two pronounced peaks and two small bumps could be identified. They evidently correspond to four hydration layers with higher local densities than the bulk water. Around the galactosyl groups one peak and a wide bump arise – implying local disorganization of hydration layers around both mono- and digalactosyl groups. Overall, galactosyl groups slightly dehydrates the membrane and the addition of MGDG results in less well-defined third hydration layer. As pointed out in the Introduction, the lipid phase behavior of thylakoid membranes strongly depends on the hydration state of membranes.

### Formations of non-bilayer structure

**Fig. 7:**
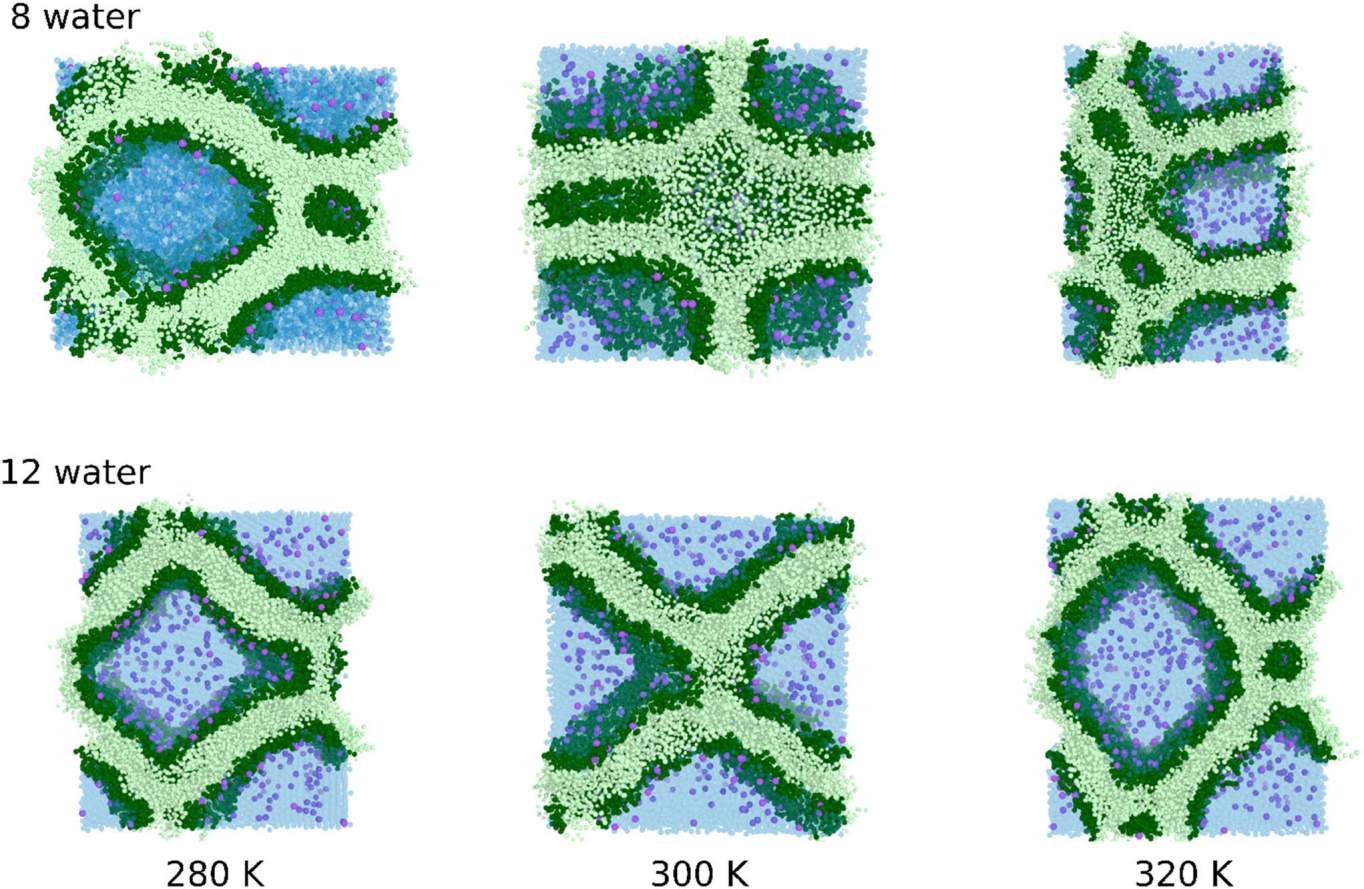
Representative snapshots of the formed non-bilayer structures of 40% MGDG containing bilayers at two hydration levels (8 water bead/lipid in first row and 12 water bead/lipid in second row) and different temperatures (280, 300 and 320 K) in first, second and third row, respectively. Dark green, light green, light blue and purple indicates the headgroups, the tails of the lipids, the water and the ions respectively. The snapshots are obtained from CG simulations

To investigate the non-bilayer forming propensity from stacked membrane pairs of thylakoid membranes with 40% MGDG content, we placed two bilayers on top of each other and conducted 8000 ns simulation. These simulations were performed at three different temperatures (280 K, 300 K, and 320 K) and two hydration levels: 12 water beads per lipid, corresponding to the full (slightly overestimated), and 8 water per lipids to moderate hydration of the membrane (Nagle and Tristram-Nagle 2000) (in an all-atom representation, these correspond to 48 and 32 water molecules per lipid, respectively). Representative snapshots are shown in Fig. 7. At all hydration levels and temperatures, non-bilayer structures were observed; however, they were not always well-defined. Interestingly, at 280 K, a well-defined inverted hexagonal structure formed at both hydration levels. This structure remained stable at higher temperatures when 12 water beads per lipid were present. However, at 8 water beads per lipid, undefined non-bilayer formations were observed at 300 K and 320 K.

**Fig. 8:**
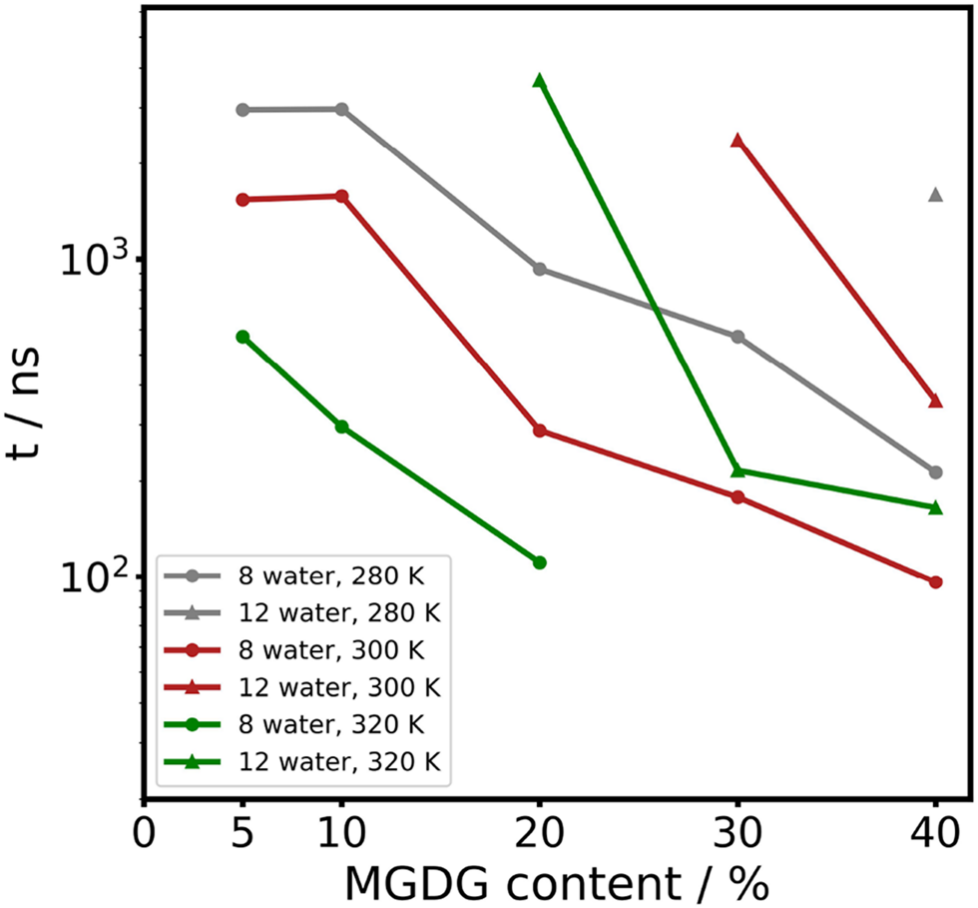
First appearance time of non-bilayer structures in CG simulations the function of MGDG content. The triangles represents the simulations with 12 water bead/lipid and the circles represents the simulations with 8 water bead/lipid. The colors correspond to different temperatures

To gain insight into the effect of MGDG content on non-bilayer formation, we conducted the same simulations as described above for a single membrane, with all MGDG concentrations. We plotted the rate of non-bilayer formation as a function of MGDG content at two different hydration levels and three different temperatures (Fig. 8). We defined the non-bilayer formation rate with the time required for the hemifusion of bilayers, following the method of Marrink et al. (Marrink and Mark 2004). We found that the rate of non-bilayer formation (either inverted hexagonal or not well-defined) depended on both the hydration level and the temperature. At 12 water beads per lipid and the lowest temperature (280 K), the hexagonal phase appears only at 40% MGDG content, corresponding to the actual thylakoid composition. When the temperature is increased to 300 K, the hexagonal phase emerges at 30% MGDG content, and at 320 K, 20% MGDG is sufficient for non-bilayer phase formation. These observations suggest that even at a high hydration level (nearly full hydration), it is physiologically realistic for non-bilayer structures to form in thylakoids at relevant temperatures. Interestingly, reducing the hydration level to 8 water beads per lipid results in the formation of non-bilayer structures at any temperature with as little as 5% MGDG. However, at 30% and 40% MGDG at 320 K, the membrane becomes unstable and collapses into an undefined lipid structure. At lower temperatures (280 K and 300 K), although the system does not collapse, it does not form a well-defined inverted hexagonal phase. Overall, these results indicate that the non-bilayer propensity of thylakoid membranes is well-established. However, to maintain a stable inverted hexagonal phase, a certain hydration level (12 water beads per lipid in this case) is required.

**Fig. 9:**
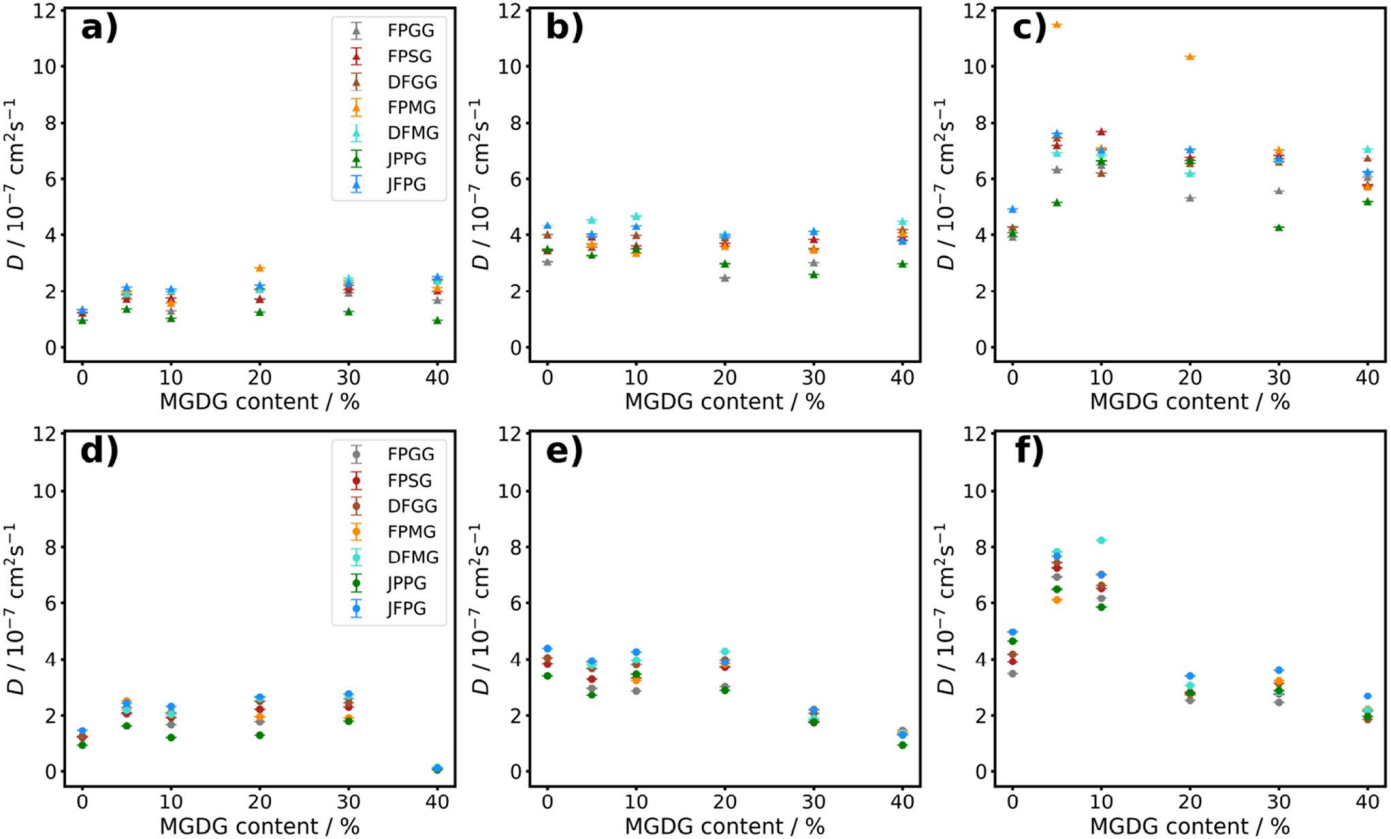
Diffusion coefficients of lipids in bilayers obtained from CG simulations at (**a**): 280 K, (**b**): 300 K and (**c**): 320 K and that of bilayer stacks at (**d**): 280 K, (**e**): 300 K and (**f**): 320 K. In case of bilayer stacks at 320 K (panel f) at 20, 30 and 40 % MGDG content inverted hexagonal phase occurs

To investigate the effect of non-bilayer formation on the mobility of thylakoid membrane lipids, we calculated the translational diffusion coefficients for each lipid at various temperatures and MGDG concentrations (Fig. 9). It is important to emphasize that we focus on informative trends rather than absolute values of the diffusion coefficients. The absolute values are not directly representative of experimental measurements due to the lack of atomistic detail in coarse-grained (CG) simulations, which tend to overestimate the diffusion coefficient (Van Eerden et al. 2017b; Ingólfsson et al. 2020). Panels a, b, and c present the diffusion coefficients of each lipid in the bilayer as a function of MGDG content. The obtained values are in agreement with the data calculated by var Eerden et al. (van Eerden et al 2015). Panels d, e, and f display the diffusion coefficients for the non-bilayer structures. As expected, the diffusion coefficients increase with temperature. Notably, within the bilayers, the lipids exhibit distinct diffusion coefficients depending on the MGDG content. Specifically, at 280 K, the diffusion coefficient decreases at 40% MGDG, while at 300 K, a reduction is observed at 30% and 40% MGDG. At 320 K, a decrease in the diffusion coefficient is seen at 20%, 30%, and 40% MGDG content, which coincides with the formation of non-bilayer structures. Thus, we can conclude that the formation of inverted hexagonal structures decreases membrane fluidity, as determined with the technique outlined above. Although there is a lack of lipid diffusion measurements in systems with compositions exactly representative of thylakoids, diffusion constants in phospholipid bilayers are documented to be of similar magnitude (Gaede and Gawrisch 2003; Gupta et al. 2019).

**Fig. 10:**
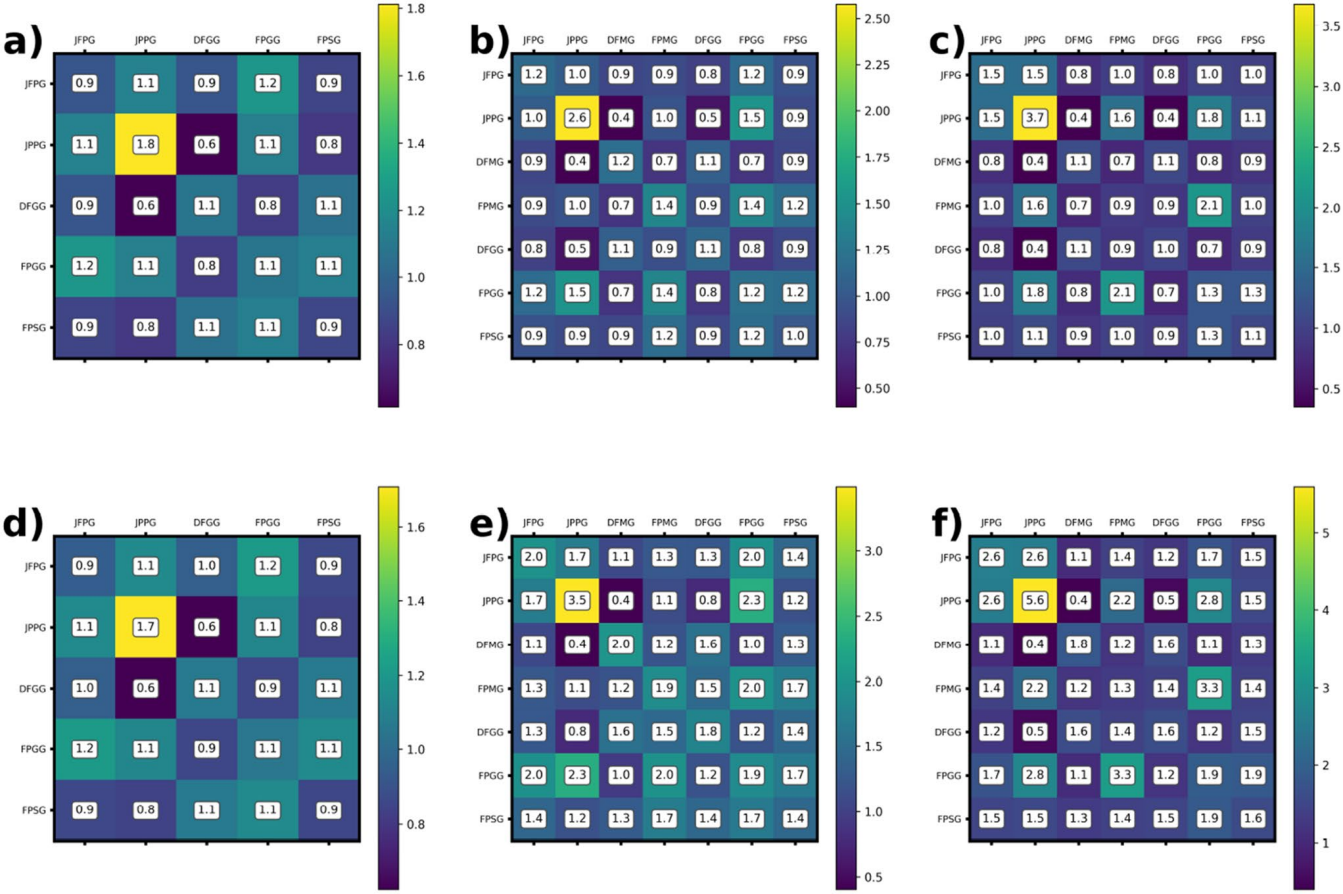
Contact matrix of bilayers obtained from CG simulations at (**a**): 0% MGDG, (**b**): 30% MGDG and (**c**): 40% MGDG content and that of stacks at (**d**): 0% MGDG, (**e**): 30% MGDG and (**f**): 40% MGDG content. All data are calculated from the simulations performed at 300 K

**Fig. 11:**
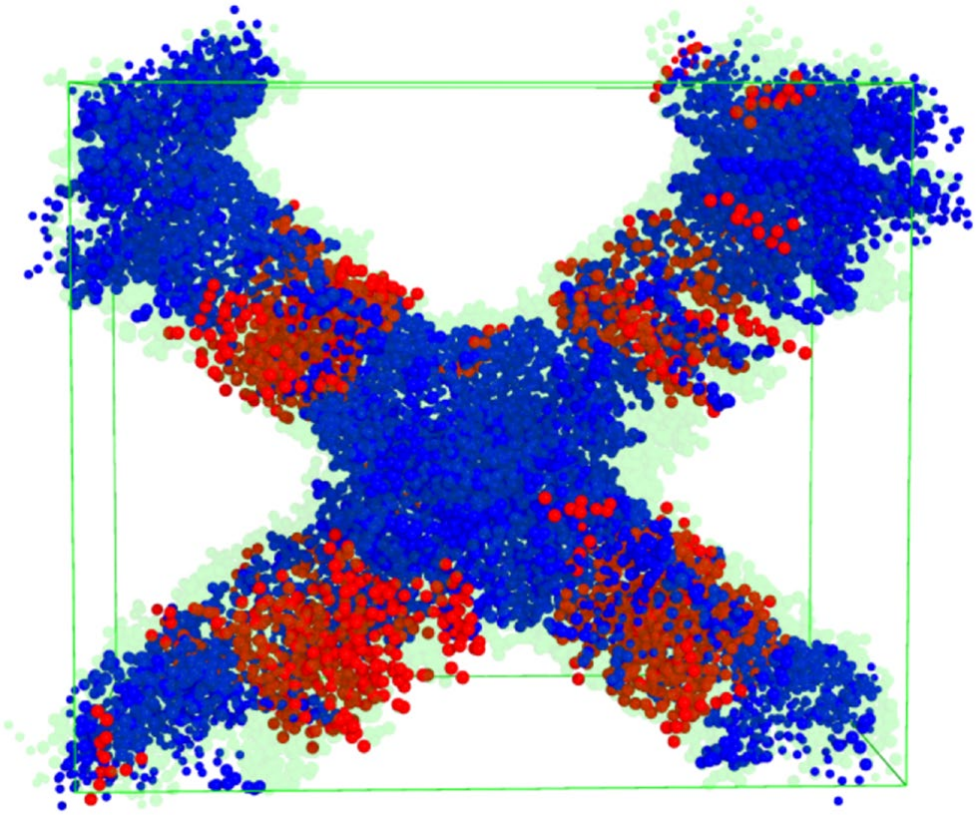
Snapshot of stack simulation with 30% MGDG at 320 K after the production run. Red lipids are the JPPGs, blue lipids are the MGDGs and light green is the whole membrane. The cube represents the unit cell. The snapshot was obtained from CG simulations

Earlier MD simulations on plant and cyanobacterial TM lipid assemblies, it has been clarified that, due to efficient lipid mixing on the microscopic scale, no large-scale phase separation occur, and only nanoscale heterogeneities appear; the formation of such lipid cluster have been shown to depend both on the headgroup charges and the saturation of the acyl chains (van Eerden et al 2015). To gain insight into lipid cluster formation, we calculated the contact matrix for 0%, 30%, and 40% MGDG content at 300 K both for bilayers and iverted hexagonal structure. The contact matrices for both the bilayers and stacks are presented in Fig. 10. Note that for 0% MGDG, no non-bilayer formation occurs (Panels a and d), whereas for 30% and 40% MGDG, the contact matrices shown in Panels e and f correspond to inverted hexagonal structures. Interestingly, in all cases, JPPG molecules exhibit clustering with themselves. There is no significant clustering of DFMG molecules is in the bilayers, while it becomes pronounced upon the formation of stacks at 30% and 40% MGDG. The same trend is observed for FPMG. Additionally, DFMG and DFGG molecules tend to migrate away from JPPG molecules in all cases. These results indicate that JPPG clustering is a general behavior of bilayers, independent of the structure. However, clustering of MGDG molecules (DFMG and FPMG) occurs only in the inverted hexagonal phase which suggests that MGDG clustering plays a role in the stabilization hexagonal structures. In Fig. 11 we show the final snapshot of inverted hexagonal phase simulation of 30% MGDG. JPPGs (red) seemingly prefer the flat region of the membrane while MGDGs (blue) appears to complement the JPPG position suggesting more preference to the curved area which is in line with the non-bilayer nature of MGDG.

## Conclusion and Perspectives

Thylakoid membranes are distinguished by their unique lipid composition, predominantly composed of galactolipids. These thylakoid lipids not only provide a scaffold for the photosynthetic membrane proteins but also significantly influence their oligomerization, long-range order, as well as their basic energy-converting and photoprotective processes (Wilhelm et al. 2020). In this study, we investigated the effects of the main, non-bilayer lipid species, monogalactosyl diacylglycerol (MGDG), on the phase behavior thylakoid lipid mixtures.

To this end, we performed all atom and coarse-grained molecular dynamics simulations of single lipid bilayers with varying MGDG concentrations in a broad range. Our results demonstrate that MGDG has a dramatic effect on membrane fluidity and the structure of these membranes. Although increasing MGDG content does not affect membrane curvature, it enhances membrane dynamics, as evidenced by increased fluctuations in membrane thickness within the bilayer. This finding adds an extra dimension to the structural dynamics of the lipid molecules in the bilayer membrane of thylakoids.

Simulations of stacked membrane pairs, using two adjacent bilayer membranes, separated by water molecules, revealed that the inclusion of MGDG leads to the formation of non-bilayer structures; and that in the absence of MGDG no such structures could be discerned. At high hydration levels (approaching full hydration of the system), at high MGDG concentrations, stable inverted hexagonal structures were observed. However, when the hydration level was decreased, the inverted hexagonal phase collapsed, indicating that an optimal hydration level is necessary to maintain this non-bilayer structure with long-range order. It is interesting to note here that desiccation of photosynthetic organisms and their resurrection upon rehydration have been shown to be associated with marked reorganizations in the TM ultrastructure (Bar Eyal et al. 2017; Mihailova et al. 2022). Well discernible ultrastructural changes have also been observed in plants exposed to long drought stress, which could be reversed by re-watering the plants (Hembrom et al. 2025). The molecular mechanisms, and the role of the non-bilayer propensity of TM lipids in particular, remain to be explored.

Our simulations also revealed that lipids exhibit preferential environments, with MGDG clustering occurring upon inverted hexagonal phase formation (see Fig. 11); however, this clustering is not a prerequisite of the self-assembly of non-bilayer structures. We also observed a decrease in lipid mobility in the inverted hexagonal state, suggesting a previously unrecognized role of non-bilayer lipid phases in modulating the diffusion of lipid molecules along the membrane.

An interesting, somewhat surprising finding of our simulations is that – at low hydration levels – very low amounts of MGDG was sufficient to trigger hemifusion. This observation raises an intriguing question beyond the case of thylakoid membranes and the energy-converting membranes with inherently high levels of non-bilayer lipids (see Introduction). In membranes, such as the plasma membranes, with much lower non-bilayer lipid contents, the ability of low amounts of non-bilayer lipids to form hemifusion channels, by displacing water molecules in the contact regions, poses the possibility of transiently formed local non-bilayer lipid phases. Our future research aims to explore this hypothesis by investigating the interplay between SNARE proteins and non-bilayer lipids in membrane fusion. We are, of course, aware of the fact that the thylakoid membranes are densely packed by membrane embedded and/or membrane-associated proteins and the area for bulk lipid molecules is restricted. Nevertheless, the phase behavior of these lipid molecules is of fundamental importance in allowing the generation and utilization of the proton motive force. In fact, it has been proposed that it is the high abundance of MGDG in TMs that, via safe-guarding the high protein density of membranes, secures the operation of the energy converting machinery based on proton-conduction pathways along protonable protein residues on the lumenal side (Kell 2024; Garg and Debnath 2025). Our findings revealing the strong dependence of the phase behavior of thylakoid lipids on their hydration state and temperature strongly suggest that the polymorphism of thylakoid lipids plays a key role in physiologically important regulatory mechanisms for example during heat and drought stresses of plants.

## Data Availability

No datasets were generated or analysed during the current study.
